# Testing for Antiphospholipid Antibody (aPL) Specificities in Retrospective “Normal” Cerebral Spinal Fluid (CSF)

**DOI:** 10.1080/10446670410001670436

**Published:** 2004-03

**Authors:** D. K. Sokol, D. R. Wagenknecht, J. A. McIntyre

**Affiliations:** 1 Department of Neurology Indiana University School of Medicine Indianapolis IN 46202 USA; 2 HLA-aPL Vascular Biology Laboratory St. Francis Hospital 1600 Albany Street Beech Grove IN 46107 USA

## Abstract

Antiphospholipid antibodies (aPL) have been found in the blood of patients with systemic and neurological disease. The rare reports of aPL in cerebral spinal fluid (CSF) have been limited mostly to IgG and IgM anticardiolipin (aCL). Our published finding of IgA aPE in the CSF of a young stroke victim prompted us to establish “normal” CSF aPL values for a panel of aPL, which included aCL, antiphosphatidylserine (aPS), antiphosphatidylethanolamine (aPE) and antiphosphatidylcholine (aPC). CSF samples were tested by ELISA for IgG, IgM and IgA aPL. In addition, the CSF samples were tested for activity in the presence and absence of phospholipid (PL) binding plasma-proteins. A total of 24 data points were obtained for each CSF sample.We tested 59 CSF samples obtained from 59 patients who were undergoing evaluation for systemic or neurologic diseases. All CSF samples had normal protein, glucose and cell counts. Ten of the 59 CSF samples (17%) had elevated aPL optical density (OD) values an order of magnitude higher than the other 49 CSF samples for one or more aPL specificity and/or isotype. One CSF sample had both PL-binding protein dependent and independent IgG aPE activity. Another CSF sample showed both IgG aPE and aPC reactivity. The remaining eight CSF samples showed single aPL findings; IgG aPE (5), IgG aPC (1), IgG aCL (1) and IgM aPC (1). Seven of 10 patients with elevated CSF values were females. As expected, most “normal” aPL OD values were substantially lower in CSF than those we have reported in blood samples from volunteer blood donors.


*Clinical & Developmental Immunology*, March 2004, Vol. 11 (1), pp. 7–12

Taylor & Francis healthsciences

### Testing for Antiphospholipid Antibody (aPL) Specificities in Retrospective "Normal" Cerebral Spinal Fluid (CSF)

D.K. SOKOL[a], D.R. WAGENKNECHT[b] and J.A. McINTYRE[b,*]

[a]*Department of Neurology, Indiana University School of Medicine, Indianapolis, IN 46202, USA;*
[b]*HLA-aPL Vascular Biology Laboratory, St. Francis Hospital, 1600 Albany Street, Beech Grove, IN 46107, USA*

Antiphospholipid antibodies (aPL) have been found in the blood of patients with systemic and neurological disease. The rare reports of aPL in cerebral spinal fluid (CSF) have been limited mostly to IgG and IgM anticardiolipin (aCL). Our published finding of IgA aPE in the CSF of a young stroke victim prompted us to establish "normal" CSF aPL values for a panel of aPL, which included aCL, antiphosphatidylserine (aPS), antiphosphatidylethanolamine (aPE) and antiphosphatidylcholine (aPC). CSF samples were tested by ELISA for IgG, IgM and IgA aPL. In addition, the CSF samples were tested for activity in the presence and absence of phospholipid (PL) binding plasma-proteins. A total of 24 data points were obtained for each CSF sample. We tested 59 CSF samples obtained from 59 patients who were undergoing evaluation for systemic or neurologic diseases. All CSF samples had normal protein, glucose and cell counts. Ten of the 59 CSF samples (17%) had elevated aPL optical density (OD) values an order of magnitude higher than the other 49 CSF samples for one or more aPL specificity and/or isotype. One CSF sample had both PL-binding protein dependent and independent IgG aPE activity. Another CSF sample showed both IgG aPE and aPC reactivity. The remaining eight CSF samples showed single aPL findings; IgG aPE (5), IgG aPC (1), IgG aCL (1) and IgM aPC (1). Seven of 10 patients with elevated CSF values were females. As expected, most "normal" aPL OD values were substantially lower in CSF than those we have reported in blood samples from volunteer blood donors.

*Keywords*: Antiphosphatidylethanolamine; Antiphosphatidylserine; Anticardiolipin; Central nervous system; Antiphosphatidylcholine

### INTRODUCTION

Antiphospholipid antibodies (aPL) in blood are associated with neurological disorders and deficits. These include focal central nervous system thrombo-occlusive events (Levine *et al.*, 2002), chorea (Paus *et al.*, 2001), migraine headaches (Silvestrini *et al.*, 1993), amnesia (Montalban *et al.*, 1989), visual abnormalities (Briley *et al.*, 1989), as well an psychosis (Chengappa *et al.*, 1991; Schwartz *et al.*, 1998) and cognitive dysfunction (Denberg *et al.*, 1997; Jacobson *et al.*, 1999). The associations of aPL with neurologic conditions other than thrombo-occlusive events have been considered "weak" and attributable to an "epiphenomenon" rather than to pathophysiologic mechanisms (Brey, 2000). A stronger association between aPL and neurological disorders might be easier to establish if aPL are sought and detected in the cerebral spinal fluid (CSF) of patients experiencing neurologic symptoms. Historically, scarce reports of aPL in CSF are limited mostly to IgG and IgM anticardiolipin (aCL) detection (Marchiorri, 1990; Lolli *et al.*, 1991; Wang *et al.*, 1992; Gallo *et al.*, 1994; Yeh *et al.*, 1994; Martinez-Cordero, 1997; Jedryka-Goral *et al.*, 2000; Lai and Lan, 2000; Baraczka *et al.*, 2002). Rarely are IgA aCL sought (Wang *et al.*, 1992). To our knowledge only one report included testing for IgG and IgM antiphosphatidylserine (aPS) and antiphosphatidylethanolamine (aPE) in CSF; the results were negative (Gallo *et al.*, 1994). Our finding of IgA aPE in the CSF of a young stroke victim (Sokol *et al.*, 2000), together with our intent to continue testing additional CSF samples, prompted us to undertake and establish "normal" aPL values for CSF. Our findings form the basis of this report.

### PATIENTS AND METHODS

Because of the potential risks associated with spinal taps to healthy individuals we were unable to obtain CSF samples from random donors. We opted to perform a retrospective analysis using residual CSF that was collected for diagnostic purposes and destined for

*Corresponding author.

ISSN 1740-2522 print/ISSN 1740-2530 online © 2004 Taylor & Francis Ltd
DOI: 10.1080/10446670410001670436

discarding after patient testing was complete. We did include, however, CSF samples from two normal elderly women who were tapped as volunteer participants in a non-related study involving Alzheimer's disease. This study was approved by the Institutional Review Board at Indiana University School of Medicine. Fifty-nine of 160 available CSF samples that met the following criteria were selected: normal protein (15–45 mg/dl), glucose (40–70 mg/dl) and cell counts ($<10/\text{cm}^3$) with a minimum volume of 2.0 ml after diagnostic testing was complete. The CSF samples were stored at 4°C until selected for evaluation whereupon the CSF were stored at −80°C until tested for aPL. Because this was a retrospective study and since the CSF samples were not released for 7 days, awaiting the possible need for further diagnostic testing, we were not able to collect blood samples for aPL testing and CSF comparisons. The patient/CSF-donor diagnoses were obtained by chart review and are listed in Table I. The mean age was 34 years (range 1–73); 26 were males and 33 females.

Our in-house serum aPL ELISA tests for IgG, IgM and IgA aPE, aPS, aCL and antiphosphatidylcholine (aPC) in the presence (dependent) and absence (independent) of supplemental PL-binding plasma proteins (McIntyre *et al.*, 2003a). A total of 24 tests were performed for each CSF sample. The ELISA was modified for CSF testing as previously published (Sokol *et al.*, 2000). Briefly, the modifications included: (1) Dilution of CSF, 1:4; (2) each dilution was tested in duplicate and (3) to ensure detection the ELISA plate wells containing CSF were developed for 2 h in the presence of substrate. Due to decreased immunoglobulin levels in CSF we used a lesser dilution than for serum aPL testing. Preliminary testing of CSF at a 1:2 dilution found that some CSF samples clotted or became gelatinous after dilution in the aPL buffers, this did not occur in dilutions of 1:4. Testing was performed in duplicate rather than in triplicate as in our serum aPL to reduce the amount of CSF required to perform the 24 aPL tests. CSF which had poor reproducibility was repeat tested as were all CSF with elevated aPL values.

TABLE I CSF donor diagnoses

| Diagnosis | Donor number |
|---|---|
| Neurological | |
| Brain tumor | 4 |
| Brain cyst | 1 |
| Dementia | 3 |
| CIPD | 1 |
| Encephalopathy | 2 |
| Headache | 9 |
| Hydrocephalus | 5 |
| Cerebellar infarct | 1 |
| Seizure | 2 |
| Multiple sclerosis | 3 |
| Involuntary movement | 1 |
| Hematological | |
| Acute lymphocytic leukemia (ALL) | 8 |
| Acute myelogenous leukemia (AML) | 4 |
| Lymphoma | 3 |
| Wegener's granuloma | 1 |
| Infectious | |
| Sepsis | 4 |
| Hepatitis C | 1 |
| HIV | 1 |
| Viral infection | 1 |
| Sarcoid | 1 |
| Mitochondrial myopathy | 1 |
| Normal volunteers | 2 |
| Total | 59 |

### RESULTS

Compared to serum samples that are tested at dilutions of 1:100, the $OD_{410}$ values obtained with CSF, diluted at 1:4, were lower than those reported for 775 normal blood donors (mean 0.013 vs. 0.028, respectively). Using the ELISA modifications described above, the $OD_{410}$ values for CSF ranged from 0.000–0.375. The graphs depicting the ELISA values inclusive of all 59 CSF samples are shown in Fig. 1. The aPL data generated are non-parametric, thus analyses by means plus or minus the standard deviations are not statistically appropriate. The small volume of CSF obtained left insufficient volume to measure total immunoglobulin G, M and A levels after aPL testing. Thus, we were unable to index our aPL isotype findings to published immunoglobulin levels for CSF. Despite the overall low mean aPL CSF value of 0.013, there were 10/59 CSF patient samples that had one or more $OD_{410}$ aPL ELISA values more than an order of magnitude above the others ($>0.150$, range: 0.150–0.375). These 10 patients' ELISA results and their final diagnoses are shown in Table II. The highest $OD_{410}$ value, 0.375 was observed for PL-binding protein independent IgG aPE and was found in the CSF obtained from an MS patient. This patient also had elevated levels of PL-binding protein dependent IgG aPE. Both IgG aPE and aPC were found in the CSF of one patient with acute myelogenous leukemia (AML). The remaining 8 positive CSF samples were positive in but one of the 24 individual aPL tests performed. Females comprised 56% of the CSF donors, however, 70% of the CSF with elevated aPL were obtained from female donors. While not significant ($P = 0.2660$, $\chi^2$ analysis), this demonstrates a trend which suggests that, as in the blood, autoantibodies detected in CSF may be more prevalent in females than in males. The CSF samples obtained from the two elderly female controls showed means of 0.011 and 0.003, respectively.

Drugs have been implicated in the appearance of the lupus anticoagulant as well as other aPL. Because of the reports linking drugs and aPL, we compiled a list of the drugs used among the 59 participants in this study. Including the over the counter (OTC) by prescription only, there were 135 different drugs tallied. Making an assumption that drugs found in the aPL negative group of 49 patients were neither associated with nor responsible for the appearance of aPL, we subtracted these drugs from the drug list compiled for the 10 aPL positive patients.

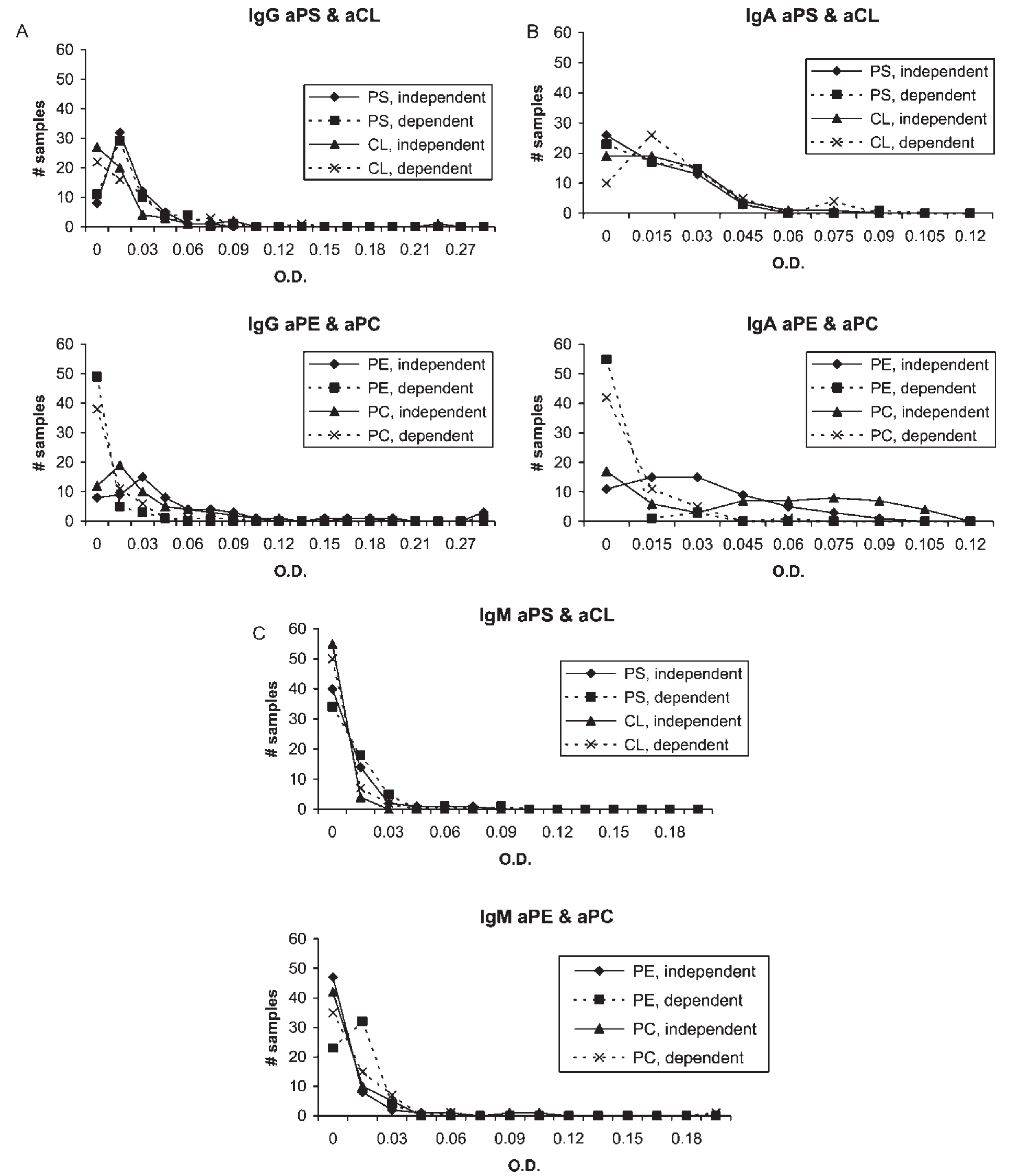

FIGURE 1 aPL findings in 59 CSF samples. Each CSF was diluted separately into dilution buffers with and without supplemental phospholipid (PL) binding plasma proteins (dependent and independent, respectively) prior to addition to PL coated ELISA wells. The distribution of ELISA readings ($OD_{410}$) for antiphosphatidylethanolamine (aPE), antiphosphatidylcholine (aPC), antiphosphatidylserine (aPS) and anticardiolipin (aCL) are shown. $OD_{410}$ findings for IgG (A), IgA (B) and IgM (C) are depicted in separate graphs.

We realize, however, that this assumption may be overreaching insofar as responses to antigenic stimuli are based upon an individual's genetic predisposition. Nevertheless, seven drugs were listed for five of the 10 aPL positive patients that were not listed among the 49 aPL negative patients. These seven are found in Table III along with the results of a literature search that sought possible associations with aPL. The published studies regarding valproate, risperidone and lamotrigine were anecdotal, conflicting and limited to serum or plasma aCL and/or lupus anticoagulant findings; none had examined CSF. Thus, there does not appear to be any reproducible

TABLE II aPL ELISA findings in "normal" CSF diluted in PL-binding protein dependent (10% adult bovine plasma) and independent (1% BSA) diluent buffers. CSF samples with elevated aPL ELISA findings are described below

| aPL | PL-binding protein requirement | Patient diagnosis | ELISA $OD_{410}$ reading |
|---|---|---|---|
| IgG aPE | Independent | Cerebellar infarct | 0.150 |
| | | Brain tumor | 0.162 |
| | | Brain cyst | 0.174 |
| | | Hepatitis C | 0.195 |
| | | AML* | 0.330 |
| | | Sarcoid | 0.338 |
| | | MS* | 0.375 |
| IgG aPE | Dependent | MS* | 0.273 |
| IgG aPC | Independent | Lymphoma | 0.153 |
| | | AML* | 0.166 |
| IgG aCL | Independent | HIV | 0.225 |
| IgM aPC | Dependent | Brain tumor | 0.180 |

* Single patients with two positive aPL.

association with these particular drugs and aPL induction. The association shown for cyclosporine was limited to bone marrow transplant recipients and their development of lupus anticoagulants. There are many other cyclosporine aPL associations in the literature, but these were indirect as the patients taking this drug were aPL positive prior to exposure.

### DISCUSSION

The presence of aPL in CSF may help to explain certain reported associations of aPL with many neurological disorders and deficits. Indeed, there is evidence that some aPL detected in the central nervous system (CNS) may be synthesized *in situ* and not result from extravasation through the CNS-associated vasculature (Martinez-Cordero, 1997; Sokol *et al.*, 2000; Baraczka *et al.*, 2002). Although there are no published reports to confirm that intrathecally-produced aPL, do not escape into the systemic circulation and vice versa, locally produced aPL in the CNS might be responsible for some aPL-associated symptoms in otherwise aPL seronegative patients (Miret *et al.*, 1997). To answer this question, paired samples of blood and CSF are needed, but this was not the objective of our present study. We undertook to establish "normal" positive/negative cutoff values for CSF aPL for future studies wherein paired blood/CSF samples will be collected for comparisons. Nonetheless, to our knowledge this is the first in-depth recording of aPL in CSF to appear. In addition to the four aPL specificities tested with three different conjugated isotype probes (IgG, IgM, IgA), we determined the PL-binding plasma protein requirement for each PL-isotype combination.

In general, elevations of aPL in our "normal" CSF samples were observed in patients with conditions previously associated with aPL, for example, infarctions (Levine *et al.*, 2002), MS and demyelinating diseases (Baraczka *et al.*, 2002), and HIV (Leder *et al.*, 2001). The association of aPL with structural brain lesions such as a tumor is not altogether surprising, as this has been reported in rare cases (Liu *et al.*, 1999). While few studies to date have looked for an association between leukemia and aPL (Stasi *et al.*, 1993; Bulvik *et al.*, 1995; Yahata *et al.*, 1997; Al-Abdulla *et al.*, 2001; McIntyre and Wagenknecht, 2001; Mitchell, 2003), there has been a report of a pro-coagulant state with presence of aPL in leukemic patients' serum peri- and post-bone marrow transplant (BMT) (Tsakiris *et al.*, 1991). Both of the patients diagnosed with leukemia in the latter study were being considered for BMT at the time of lumbar puncture.

The importance of aPL detection in the presence and absence of PL-binding plasma proteins is gaining acceptance. Recently, there have been several papers documenting the pathogenic effects of plasma protein independent aPS on pregnancy associated tissues. Direct binding of monoclonal aPS to human trophoblast, inhibition of hCG production and blocking trophoblast invasiveness have been observed for plasma protein independent aPS (Katsuragawa *et al.*, 1997; Di Simone *et al.*, 2000). The aPE we observed in the CSF of a 15-year-old stroke patient was plasma protein independent (Sokol *et al.*, 2000), and we have shown that certain aPL associated with early rejection of solid organ grafts were independent of plasma proteins (McIntyre and Wagenknecht, 2003). Indeed, the majority of CSF aPL described in this report 10/12 were classified as plasma protein independent. Had we not screened the samples independent of supplemental plasma proteins, these 10 aPL specificities would have gone missing. Although we cannot attribute pathology to these CSF samples, the possibility remains intriguing, especially knowing that the tissues comprising the CNS are rich sources of potential PL-binding protein independent aPL targets.

TABLE III Drugs unique to 5 aPL-positive patients

| Drug name (generic) | aPL association | Reference(s) |
|---|---|---|
| Percocet (oxycodone/acetaminophen) | No | None |
| Fosamax (alendronate sodium)* | No | None |
| Depakene (valproic acid)* | Yes/No | Furmaga *et al.*, 1997; Echaniz-Laguna *et al.*, 1999 |
| Risperdal (risperidone)* | Yes/No | Sarzi-Puttini *et al.*, 2000; Kamijo *et al.*, 2003 |
| Lamictal (lamotrigine) | Yes/No | Furmaga *et al.*, 1997; Echaniz-Laguna *et al.*, 1999 |
| Morphine Sulfate | No | None |
| Neoral (cyclosporine) | Yes | Greeno *et al.*, 1995 |

* The same patient for these 3 drugs.

Drug-induced aPL associations have been the subject of several publications (reviewed in Haag and Spigset, 2002). There are conflicting data regarding drug-associated aPL and pathology with some authors concluding that drug-induced aPL are not associated with thrombosis (Pardo *et al.*, 2001) while others report the frequency of complications are the same as what is seen with the autoimmune patients (Triplett *et al.*, 1988). Considering that the drugs listed in Table III were unique to the putative aPL positive CSF samples, there were conflicting data presented for valproic acid, risperidone and lamotrigine. While some investigators directly implicated the finding of lupus anticoagulant and aCL to these drugs (Furmaga *et al.*, 1997; Echaniz-Laguna *et al.*, 1999), others found no association (Sarzi-Puttini *et al.*, 2000; Kamijo *et al.*, 2003). Lupus inhibitors did appear in bone marrow transplant recipients subsequent to cyclosporine exposure, but these patients were also exposed to many other immunosuppressive drugs and therapies (Greeno *et al.*, 1995).

There are several caveats to relate regarding these drug-associated aPL studies that might account for the discrepancies. First, the methodology used for aPL detection varied among the reporting laboratories. Some investigators limited their aPL analyses to IgG only whereas others reported IgG and IgM. Second, the choices of buffers and proteins included in the buffer diluents were different. Third, there has been and remains an historical problem with standardization of the ELISA used for aPL detection. Many of the problems that confound aPL testing and reporting have been presented and discussed in a recent review (McIntyre *et al.*, 2003b).

In summary, we have provided a basis for comparisons of aPL activity in CSF. Nonetheless, we recommend that patient history be carefully scrutinized for conditions and/or occurrences with known aPL associations before proceeding with studies of CSF aPL. Serum and CSF comparisons, and when possible, CNS tissue samples would be important to further understand the association between aPL and neurologic disease.

### Acknowledgements

We thank Ms Benita Book, Department of Surgery for assistance with this study.

### References

Al-Abdulla, N.A., Thompson, J.T. and LaBorwit, S.E. (2001) "Simultaneous bilateral central retinal vein occlusion associated with anticardiolipin antibodies in leukemia", *Am. J. Opthalmol.* **132**, 266–268.

Baraczka, K., Lakos, G. and Sipka, S. (2002) "Immunoserological changes in the cerebro-spinal fluid and serum in systemic lupus erythematosus patients with demylinating syndrome and multiple sclerosis", *Acta Neurol. Scan* **105**, 378–383.

Brey, R.L. (2000) "Differential diagnosis of central nervous system manifestations of the Antiphospholipid antibody syndrome", *J. Autoimmun.* **15**, 133–138.

Briley, D.P., Coull, B.M. and Goodnight, S.H. (1989) "Neurological disease associated with antiphospholipid antibodies", *Ann. Neurol.* **25**, 221–227.

Bulvik, S., Aronson, I., Ress, S. and Jacobs, P. (1995) "Extensive bone marrow necrosis associated with antiphospholipid antibodies", *Am. J. Med.* **98**, 572–574.

Chengappa, K.N., Carpenter, B., Keshavan, M.S., Yang, Z.W., Kelly, R.H., Rabin, B.S. and Ganguli, R. (1991) "Elevated IgG and IgM anticardiolipin antibodies in a subgroup of medicated and unmedicated schizophrenic patients", *Biol. Psychiatr.* **30**, 731–735.

Denberg, S.D., Carbotte, R.M., Ginsberg, J.S. and Denberg, J.A. (1997) "The relationship of antiphospholipid antibodies to cognitive function in patients with systemic lupus erythematosus", *J. Int. Neuropsychol. Soc.* **3**, 377–386.

Di Simone, N., Meroni, P.L., del Papa, N., Raschi, E., Caliandro, D., De Carolis, C.S., Khamashta, M.A., Atsumi, T., Hughes, G.R., Balestrieri, G., Tincani, A., Casali, A. and Caruso, A. (2000) "Antiphospholipid antibodies affect trophoblast gonadotropin secretion and invasiveness by binding directly and through adhered beta2-glycoprotein I", *Arthritis Rheum.* **43**, 140–150.

Echaniz-Laguna, A., Thiriaux, A., Ruolt-Olivesi, I., Marescaux, C. and Hirsch, E. (1999) "Lupus anticoagulant induced by combination of valporate and lamotrigine", *Epilepsia* **40**, 1661–1663.

Furmaga, K.M., De Leon, O.A., Sinha, S.B., Jobe, T.H. and Gaviria, M. (1997) "Psychosis in medical conditions: response to risperidone", *Gen. Hosp. Psychiatr.* **19**, 223–228.

Gallo, P., Sivieri, S., Ferrarini, A.M., Giometto, B., Ruffatti, A., Ritter, E., Chizzolini, C. and Tavolato, B. (1994) "Cerebrovascular and neurological disorders associated with antiphospholipids in CSF and serum", *J. Neurol. Sci.* **122**, 97–101.

Greeno, E.W., Haake, R., McGlave, P., Weisdorf, D. and Verfaillie, C. (1995) "Lupus inhibitors following bone marrow transplant", *Bone Marrow Transplant.* **15**, 287–291.

Haag, S. and Spigset, O. (2002) "Antipsychotic-induced venous thromboembolism: a review of the evidence", *CNS Drugs* **16**, 765–776.

Jacobson, M.W., Rapport, L.J., Keeman, P.A., Coleman, R.D. and Tietjen, G.E. (1999) "Neuropsychological deficits associated with antiphospholipid antibodies", *J. Clin. Exp. Neuropsychol.* **21**, 251–264.

Jedryka-Goral, A., Zabek, J., Wojciechowska, B., Zaborski, J., Chwalinska-Sadowska, H. and Czlonkowska, A. (2000) "Evaluation of cerebrospinal fluid for the presence of anticardiolipin antibodies (aCL) in NP-SLE patients", *Clin. Rheumatol.* **19**, 306–310.

Kamijo, Y., Soma, K., Nagai, T., Kurihara, K. and Ohwada, T. (2003) "Acute massive pulmonary thromboembolism associated with risperidone and conventional phenothiazines", *Circ. J.* **67**, 46–48.

Katsuragawa, H., Kanzaki, H., Inoue, T., Hirano, T., Mori, T. and Rote, N.S. (1997) "Monoclonal antibody against phosphatidylserine inhibits *in vitro* human trophoblastic hormone production and invasion", *Biol. Reprod.* **56**, 50–58.

Lai, N-S. and Lan, J-L. (2000) "Evaluation of cerebrospinal anti-cardiolipin antibodies in lupus patients with neuropsychiatric manifestations", *Lupus* **9**, 353–357.

Leder, A.N., Flansbaum, B., Zandman-Goodard, G., Asherson, R. and Shoenfeld, Y. (2001) "Antiphospholipid syndrome induced by HIV", *Lupus* **10**, 370–374.

Levine, J.S., Branch, W.D. and Rauch, J. (2002) "Medical progress: the antiphospholipid syndrome", *N. Engl. J. Med.* **346**, 752–763.

Liu, E., Robertson, R.L., du Plessis, A. and Pomeroy, S.L. (1999) "Basal ganglia germinoma with progressive cerebral hemiatrophy", *Pediatr. Neurol.* **20**, 312–314.

Lolli, F., Mata, S., Baruffi, M.C. and Amaducci, L. (1991) "Cerebrospinal fluid anti-cardiolipin antibodies in neurological diseases", *Clin. Immunol. Immunopathol.* **59**, 314–321.

Marchiorri, P.E., Dos Reis, M., Quevedo, M.E., Callegaro, D., Hirata, M.T., Scaff, M. and De Oliveira, R.M. (1990) "Cerebrospinal fluid and serum antiphospholipid antibodies in multiple sclerosis, Guillian–Barre syndrome and systemic lupus erythematosus", *Arg. Neuropsiquiatr* **48**, 465–468.

Martinez-Cordero, E., Rivera Garcia, B.E. and Aguilar Leon, D.E. (1997) "Anticardiolipin antibodies in serum and cerebrospinal fluid from patients with systemic lupus erythematosus", *J. Investig. Allergol. Clin. Immunol.* **7**, 596–601.

McIntyre, J.A. and Wagenknecht, D.R. (2001) "Antiphospholipid antibodies: risk assessments for solid organ, bone marrow, and tissue transplantation", *Rheum. Dis. Clin. N. Am.* **27**, 611–631.

McIntyre, J.A. and Wagenknecht, D.R. (2003) “Antiphospholipid antibodies and renal transplantation: a risk assessment”, *Lupus* **12**, 555–559.

McIntyre, J.A., Wagenknecht, D.R. and Waxman, D.W. (2003a) “Frequency and specificities of antiphospholipid antibodies (aPL) in volunteer blood donors”, *Immunobiology* **207**, 59–63.

McIntyre, J.A., Wagenknecht, D.R. and Faulk, W.P. (2003b) “Antiphospholipid antibodies: discovery, definitions, detection and disease”, *Prog. Lipid Res.* **42**, 176–237.

Miret, C., Cervara, R., Reverter, J.C., Garcia-Carrasco, M., Ramos, M., Font, J. and Ingelmo, M. (1997) “Antiphospholipid syndrome without antiphospholipid antibodies at the time of the thrombotic event: transient ‘seronegative’ antiphospholipid syndrome?”, *Clin. Exp. Rheumatol.* **15**, 541–544.

Mitchell, L.G. (2003) “A prospective cohort study determining the prevalence of thrombotic events in children with acute lymphoblastic leukemia and a central venous line who are treated with L-asparaginase”, *Cancer* **97**, 508–516.

Montalban, J., Arboux, A., Staub, H., Barquinero, J., Marti-Vilalta, J., Codina, A. and Hughes, G.R.V. (1989) “Transient global amnesia and antiphospholipid antibodies”, *Clin. Exp. Rheumatol.* **7**, 85–87.

Pardo, A., Gonzalez-Porque, P., Gobernado, J.M., Jimenez-Escrig, A. and Lousa, M. (2001) “Study of antiphospholipid antibodies in patients treated with antiepileptic drugs”, *Neurologica* **16**, 7–10.

Paus, S., Potzsch, B., Risse, J.H., Klockgether, T. and Wullner, U. (2001) “Chorea and antiphospholipid antibodies: treatment with methotrexate”, *Neurology* **56**, 137–138.

Sarzi-Puttini, P., Panni, B., Cazzola, M., Muzzupappa, S. and Turiel, M. (2000) “Lamotrigine-induced lupus”, *Lupus* **9**, 555–557.

Schwartz, M., Rochas, M., Weller, B., Sheinkman, M.D., Tal, I., Golan, D., Toubi, N., Eldar, I., Sharf, B. and Attias, D. (1998) “High association of anticardiolipin antibodies with psychosis”, *J. Clin. Psychiatr.* **59**, 20–23.

Silvestrini, M., Letizia, L.M., Matteis, M., DeSimone, R. and Bernardi, G. (1993) “Migraine in patients with stroke and antiphospholipid antibodies”, *Headache* **33**, 421–426.

Sokol, D.K., McIntyre, J.A., Short, R.A., Gutt, J., Wagenknecht, D.R., Biller, J. and Garg, B. (2000) “Henoch-Schönlein purpura and stroke: antiphosphatidylethanolamine antibody in CSF and serum”, *Neurology* **55**, 1379–1381.

Stasi, R., Stipa, E., Masi, E., Oliva, F., Sciarra, A., Perrotti, A., Zaccari, G. and Papa, G. (1993) “Antiphospholipid antibodies: prevalence, clinical significance and correlation to cytokine levels in acute myeloid leukemia and non-Hodgkin’s lymphoma”, *Thromb. Haemostasis.* **18**, 568–572.

Triplett, D.A., Brandt, J.T., Musgrave, K.A. and Orr, C.A. (1988) “The relationship between lupus anticoagulants and antibodies to phospholipids”, *J. Am. Med. Assoc.* **259**, 550–554.

Tsakiris, D.A., Huser, B., Gratwohl, A. and Marbet, G.A. (1991) “Tendency to thrombosis following bone marrow transplantation?”, *J. Suisse Med.* **121**, 341–343.

Wang, C-R., Chuang, C-Y. and Chen, C-Y. (1992) “Anticardiolipin antibodies and interleukin-6 in cerebrospinal fluid and blood of Chinese patients with neuro-Behcet’s syndrome”, *Clin. Exp Rheumatol.* **10**, 599–602.

Yahata, N., Ohyashiki, K., Iwama, H., Katagiri, T., Kodama, S., Tauchi, T., Yaguchi, M. and Toyama, K. (1997) “Chronic myelomonocytic leukemia in a patient with antiphospholipid syndrome: first case report”, *Leuk. Res.* **21**, 889–890.

Yeh, T.-S., Wang, C.-R., Jeng, G.-W., Lee, G.-L., Chen, M.-Y., Wang, G.-R., Lin, K.-T., Chuang, C.-Y. and Chen, C.-Y. (1994) “The study of anticardiolipin antibodies and interleukin-6 in cerebrospinal fluid and blood of Chinese patients with systemic lupus erythematosus and central nervous system involvement”, *Autoimmunity* **18**, 169–175.

